# A significant change towards cemented fixation in revision total hip arthroplasty in patients younger than 55 years in the Netherlands: results of an observational cohort study in the Dutch Arthroplasty Register in 28,516 primary hip replacements and 1285 revision procedures

**DOI:** 10.1177/11207000211020002

**Published:** 2021-06-08

**Authors:** Martijn FL Kuijpers, Gerjon Hannink, Liza N van Steenbergen, B Wim Schreurs

**Affiliations:** 1 Department of Orthopaedics, Radboud Institute for Health Sciences, Radboud University Medical Centre, Nijmegen, The Netherlands; 2 Department of Operating Rooms, Radboud Institute for Health Sciences, Radboud University Medical Centre, Nijmegen, The Netherlands; 3 Dutch Arthroplasty Register (Landelijke Registratie Orthopedische Implantaten), ‘s-Hertogenbosch, The Netherlands

**Keywords:** Fixation method, revisions, total hip arthroplasty, young patients

## Abstract

**Background:**

Worldwide, the majority of total hip arthroplasties (THAs) placed in patients <55 years are uncemented. However, little is known about the preferred method of fixation in revision hip arthroplasty in young patients. The aim of this study was to assess potential differences in the method of fixation used between primary and revision THA in young patients using data from the Dutch Arthroplasty Register.

**Methods:**

All primary THA placed in patients younger than 55 years, registered in the LROI between 2007 and 2019 were included *n* = 28,516). Kaplan-Meier survival analyses were used to estimate the survival of primary THA by method of fixation. Additionally, survival of revision procedures that changed or did not change in method of fixation were estimated. McNemar’s test was used to assess differences in the proportion of cemented and uncemented fixation between primary and revision THA.

**Results:**

In all acetabular revisions, the use of cemented fixation increased statistically significant with 39% (95% CI, 34–45, *p* < 0.001) from 23% in primary THA to 62% in revision procedures. In all femoral revisions, the increase of cemented fixation was also statistically significant with 25% (95% CI, 19–31, *p* < 0.001), from 11% in primary THA to 36% in revision surgery. For both revised acetabular and femoral components, we found no statistically significant difference in the 5-year survival between revision procedures that changed or did not change in method of fixation.

**Conclusions:**

There was a significant change towards cemented fixation between primary and revision THA in young patients in the Netherlands, which was especially pronounced in acetabular revisions. No significant difference in short-term survival was found between revision procedures that changed or did not change in method of fixation. Long-term follow-up data are needed to evaluate the effect of this change in fixation method on the outcome of revision procedures in young patients.

## Introduction

In recent years, the number of total hip arthroplasties (THAs) placed in young patients has increased. Projections show that in 10 years, more than half of all primary THA will be placed in patients <65 years, with the biggest increase expected in patients between 45 and 55 years of age.^1,2^ Unfortunately, registry data show lower survival rates of primary THA in these younger patients when compared to patients >55 years.^3–5^ Due to this lower survival, in combination with the increase of the number of THA performed, an increase in revision hip arthroplasty in younger patients is inevitable.^1,6,7^

Remarkably, there is little data available on the outcome of revision procedures in young patients. To our knowledge, there are only 2 papers on the long-term outcome of revision THA in patients >55 years at the time of their revision. 1 study reported an alarming survival rate of 63% at 10 years using uncemented implants,^8^ where the other, using a biological reconstruction technique with impaction bone grafting and cemented THA, showed more promising results with a 10-year survival rate of 87%.^9^

In addition, there is little information available on the fixation techniques used in revision procedures in these young patients. As in most countries, the majority of primary THA in the Netherlands are placed using uncemented techniques.^10^ However, there is no information available based on registry data on the method of fixation used in revision procedures in young patients.

In this study we included a large cohort of primary THA and their subsequent revision procedures in patients <55 years at the moment of primary THA from the Dutch Arthroplasty Register (LROI). The aim of this study was to assess differences in the method of fixation used between primary and revision hip arthroplasty in young patients using data from the LROI.

## Methods

The Dutch Arthroplasty Register was initiated by the Netherlands Orthopaedic Association (NOV) in 2007. It is a nationwide, population-based register, collecting data about joint arthroplasty in the Netherlands. Coverage of all Dutch hospitals was achieved in 2012. Completeness of the register reached 99% in 2019 for primary THA, and 97% of revision hip arthroplasty.^3^ Prostheses characteristics are derived from an implant library within the LROI, where all characteristics of prostheses used in the Netherlands are available.^11^

For the present study, we included all primary THAs performed in patients <55 years, registered in the LROI between 01 January 2007 and 31 December 2019. Exclusion criteria for primary procedures were hip resurfacings and primary THA inserted for oncological reasons. We then extracted all subsequent revision procedures from this cohort to study the method of fixation in primary and revision THA.

Within the LROI, a revision procedure is defined as an exchange of at least 1 of the components of the prosthesis. 3 different types of revision procedures are distinguished: (1) a total revision, where both the acetabular and femoral part of the implant are exchanged; (2) a major partial revision procedure, where the acetabular or the femoral component of the implant is exchanged; (3) a minor partial revision, where only the head and/or insert of the implant is exchanged.

In the LROI, fixation method is registered as uncemented, cemented, hybrid (cemented stem, uncemented cup), or reversed hybrid fixation (uncemented stem, cemented cup). Therefore, acetabular components were defined as cemented when registered as part of a completely cemented THA or registered as part of a reversed hybrid in primary and revision THA and defined as uncemented when registered as part of a complete uncemented THA or as part of a hybrid THA. Femoral components were defined as cemented when registered as part of a complete cemented THA or as part of a hybrid THA and defined as uncemented when registered as part of a complete uncemented THA or as part of a reversed hybrid THA.

### Ethics

Ethical approval was not required as all data were received completely anonymous. Data are available from the LROI (Dutch Arthroplasty Registry), but restrictions apply to the availability of these data, which were used under licence for the current study.

### Statistics

Baseline characteristics of all included patients were provided. Continuous variables were described using means and standard deviations (SDs), or median and interquartile range, where appropriate. Categorical data were described using counts and percentages. McNemar’s test was used to assess differences in the proportion of cemented and uncemented fixation between primary and revision hip arthroplasty. Differences in proportions were reported including their 95% confidence intervals (CI). Confidence intervals accounting for matched nature of the data were calculated as described by Agresti and Min.^12^ Level of significance was set at *p* < 0.05.

Using Kaplan-Meier survival analyses, survival rates by method of fixation were estimated at 5 and 10 years follow-up. Survival time of primary THA was calculated as time between the moment of implantation and revision procedure, death of the patient, or the end of study follow-up (31 December 2019). Additionally, Kaplan-Meier survival analyses were used to determine the survival at 5 years follow-up of revision procedures that changed or did not change of method of fixation.

All analyses were performed using R version 3.5.1 (R Foundation for Statistical Computing, Vienna, Austria).

## Results

### Primary THA

In total, 28,516 primary THAs in patients <55 years were registered in the LROI between 01 January 2007 and 31 December 2019. More women received a THA (53.5%), median age at time of primary THA was 50 years (range 11–54 years). Other patient characteristics are presented in Table 1.

**Table 1.** Patient characteristics of 28,516 primary THA.

	*n* (%)
Age in years (median)	50 (11–54)
Gender	
Male	13,275 (46.6)
Female	15,210 (53.3)
ASA	
I	12,545 (44.0)
II	12,908 (45.3)
III–IV	2,187 (7.7)
Fixation	
Cemented	2,654 (9.3)
Uncemented	22,785 (79.9)
Reversed hybrid	2,206 (7.7)
Hybrid	586 (2.1)
Bearing type	
C-PE	14,696 (51.5)
CoC	3,391 (11.9)
M-PE	5,154 (18.1)
MoM	939 (3.3)
Zr-PE	1,952 (6.8)
Surgical approach	
Posterolateral	16,934 (59.4)
Anterior	4,788 (16.8)
Direct lateral	4,685 (16.4)
Anterolateral	1,551 (5.4)
Head diameter	
22–88 mm	7,172 (25.2)
32 mm	13,144 (46.1)
36 mm	6,143 (21.5)
⩾38 mm	811 (2.8)

C-PE: ceramic-on-polyethylene; MoM: metal-on-metal; CoC: ceramic-on-ceramic; M-PE: metal-on-polyethylene; Zr-PE: oxidised zirconium-on-polyethylene.

The most common reason for primary THA was osteoarthritis (67%), followed by dysplasia (10%) and osteonecrosis (9%). Most primary THA were placed as full uncemented (80%), followed by full cemented (9%), reversed hybrid (8%) and hybrid fixation (2%). Mean follow-up of primary THA was 5.2 years (SD 3.4).

**Figure 1.**
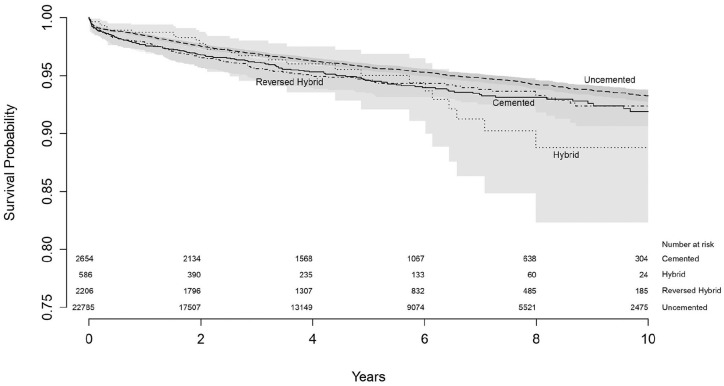
Survival of all primary THA by fixation method with endpoint revision of any reason.

Using Kaplan-Meier, the survival of all primary THA was determined for different fixation methods. The survival at 10 years follow-up for cemented THA was 91.9% (95% CI, 90.2–93.3), for uncemented THA 93.3% (95% CI, 92.7–93.7), for hybrid THA 88.8% (95% CI, 82.3–93.0) and for reversed hybrid 92.4% (95% CI, 90.6–93.8) (Table 2) (Figure 1).

**Table 2.** Survival of all primary THA by method of fixation.

Method of fixation	5-year	10-year
Cemented	94.6 (93.5–95.5)	91.9 (90.2–93.3)
Uncemented	95.7 (95.4–96.0)	93.3 (92.7–93.7)
Hybrid	95.0 (92.1–96.9)	88.8 (82.3–93.0)
Reversed hybrid	94.6 (93.5–95.6)	92.4 (90.6–93.8)

### Revision THA

There were 1285 revision procedures registered within the study follow-up. The most common reason for revision was dislocation (21%), followed by femoral loosening (18%) and infection (18%) (Table 3).

**Table 3.** Reason for revision.

	All revisions (1285)	Only cup revisions (439)	Only stem revisions (303)
Infection	236	4	7
Wear	72	41	4
Periprosthetic fracture	93	10	61
Dislocation	269	134	39
Girdlestone	40	1	0
Femoral loosening	237	1	176
Acetabular loosening	203	151	4
PAO	27	8	8
MoM	84	55	0
Other	288	127	56

PAO: periarticular ossification; MoM: symptomatic metal-on-metal.

In 284 cases, there was no revision of the acetabular and/or femoral component. These cases involved revision of the femoral head and/or insert of the implant (minor partial revision, *n* = 252), were registered as other type of revision (*n* = 20) or were missing (*n* = 12). These cases were excluded from further analysis as the fixation of the cup or stem did not change during the procedure.

Therefore, 1001 procedures involved a revision of the acetabular component and/or the femoral component. In an additional 77 cases, there was no re-implantation of the acetabular and femoral component, as these were registered as Girdlestone procedure. These cases were also excluded from further analysis, resulting in 924 revision procedure with an exchange of the acetabular and/or femoral component.

**Figure 2.**
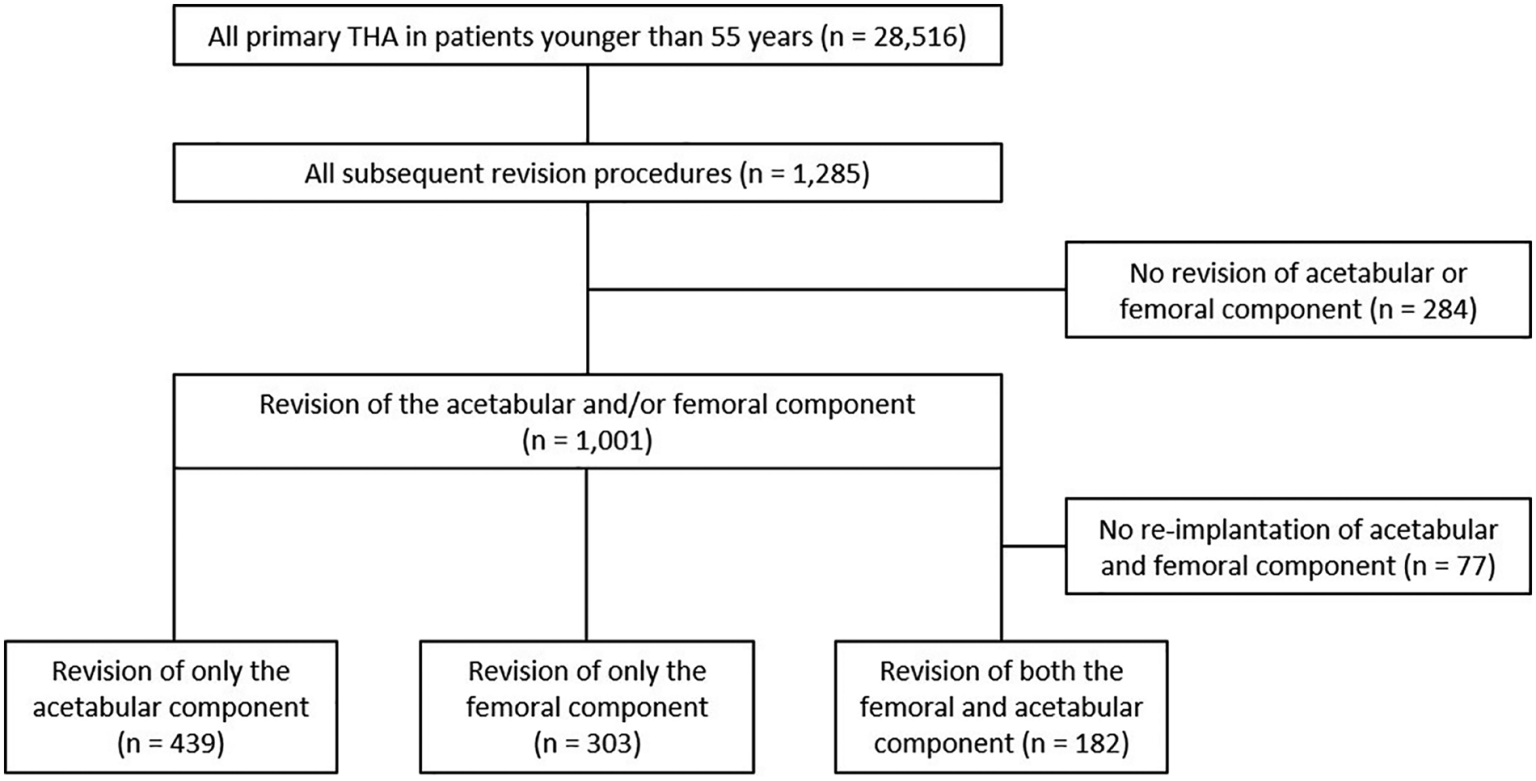
Flowchart of all included total hip replacements.

A total revision, replacing both the acetabular and femoral component, was performed in 182 cases. A major partial revision, replacing the acetabular component or femoral component, was performed in 742 cases. In 439 of those cases the acetabular component was revised, where in 303 of those cases the femoral one (Figure 2).

The most common reason for a total revision was infection (*n* = 53), followed by femoral loosening (*n* = 50). The most common reason for revision of only the acetabular component was acetabular loosening (*n* = 151), followed by dislocation (*n* = 134). For revision of only the femoral component, the most common reason was femoral loosening (*n* = 176), followed by periprosthetic fracture (*n* = 61).

### Type of fixation used in total revisions

**Figure 3.**
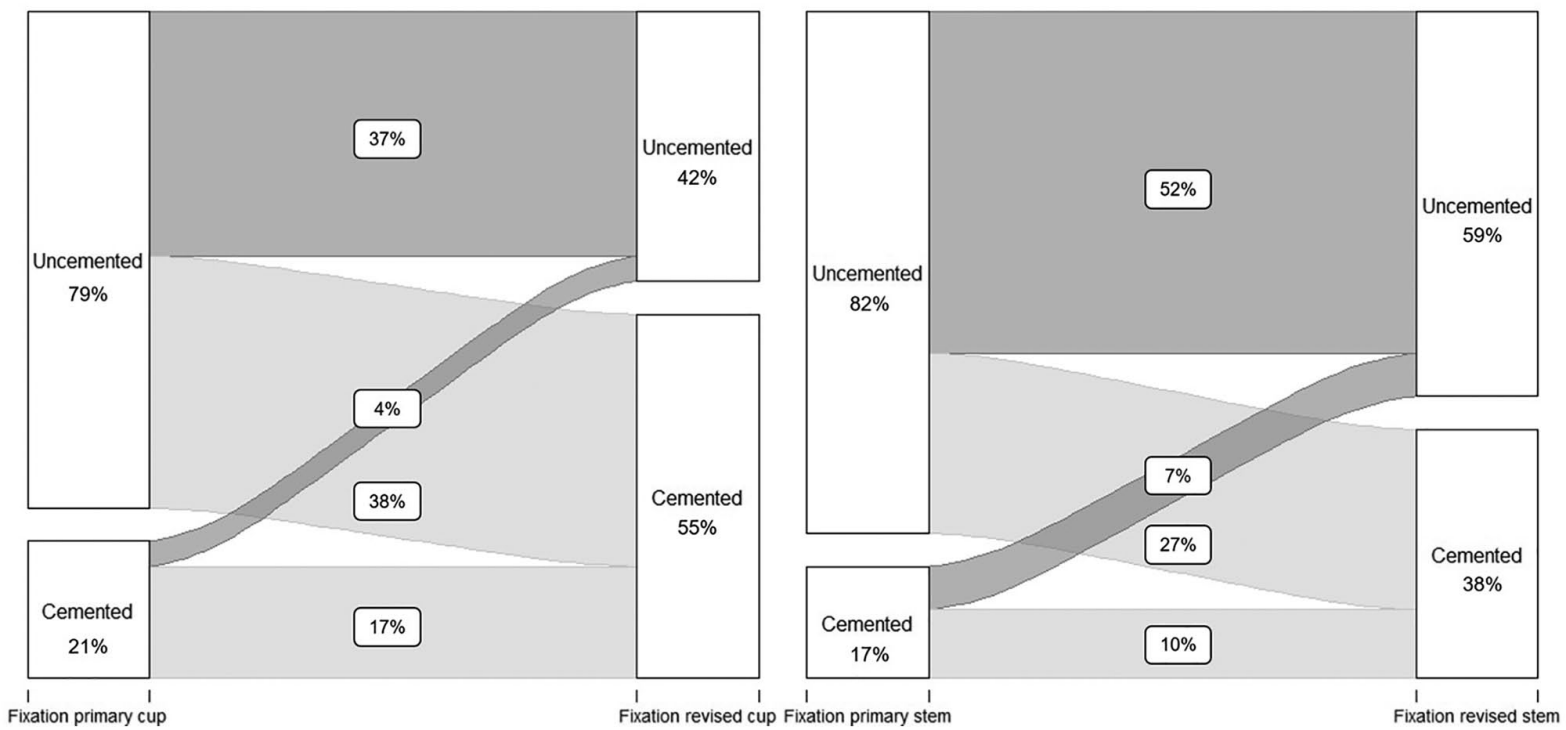
Change in fixation method in all total revisions, with acetabular fixation of total revisions on the left and femoral fixation of all total revisions on the right. Percentages do not add up to 100%, as cases with missing method of fixation are not shown in this Figure.

From the 182 total revision procedures, primary fixation was cemented in 27 cases (15%), uncemented in 139 cases (76%), hybrid in 4 (2%) and reversed hybrid in 11 (6%). In 1 case, registration of the primary method of fixation was missing. Fixation method changed from uncemented to cemented in revision procedures in 65 cases (36%). Uncemented fixation was used in 72 cases (40%), hybrid in 4 (2%) and reversed hybrid in 36 cases (20%). In 5 cases, method of fixation used for the revision procedure was missing (Table 4). Therefore, primary acetabular components were cemented in 38 cases (21%) and uncemented in 143 cases (79%). The proportion of cemented fixation of acetabular components in total revisions procedures increased statistically significantly with 36% (CI 28–44; *p* < 0.001), where 101 cups (55%) were cemented, compared to 76 uncemented cups (42%) (Figure 3).

**Table 4.** Method of fixation in primary and revision THA for total revisions (*n* = 182).

Primary	Revision					
	Cemented	Uncemented	Hybrid	Reversed hybrid	Missing	Total
Cemented	17	4	1	5	0	27
Uncemented	41	62	3	28	5	139
Hybrid	1	3	0	0	0	4
Reversed hybrid	6	2	0	3	0	11
Missing	0	1	0	0	0	1
Total	65	72	4	36	5	182

For femoral components, a similar trend was seen. Primary stems were cemented in 31 cases (17%), and uncemented in 150 cases (82%). In revision procedures, the use of cemented fixation for femoral components increased statistically significantly with 22% (CI 13–30; *p* < 0.001), with 69 cemented stems (38%), compared to 108 uncemented cases (59%) (Figure 3).

### Fixation of acetabular component

**Figure 4.**
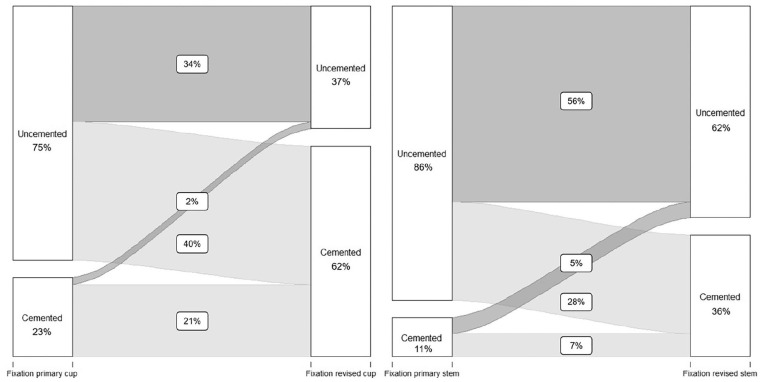
Change in fixation method for all acetabular revisions (left) and femoral revisions (right) between primary and revision THA. Percentages do not add up to 100%, as cases with missing method of fixation are not shown in this Figure.

In 439 revision procedures, only the acetabular component was revised. From these procedures, 328 cups (75%) were uncemented in primary THA (registered as uncemented and hybrid), whereas 103 (23%) were placed using bone cement (registered as cemented a reversed hybrid). Again, the fixation method changed towards cemented fixation in revision surgery. In total, 273 revised cups (62%) were fixated using bone cement (registered as cemented or reversed hybrid), and only 161 (37%) revised cups were fixated without cement (registered as uncemented or hybrid), resulting in a clear change towards cemented fixation when only the acetabular component was revised (Table 5). The proportion of cemented fixation between primary and revision THA in acetabular revision procedures increased statistically by 39% (CI 34–45, *p* < 0.001) (Figure 4).

**Table 5.** Method of fixation in primary and revision THA for cup revisions (*n* = 439).

Primary	Revision					
	Cemented	Uncemented	Hybrid	Reversed hybrid	Missing	Total
Cemented	46	3	0	3	0	52
Uncemented	155	144	1	19	3	322
Hybrid	3	3	0	0	0	6
Reversed hybrid	42	6	0	1	2	51
Missing	4	4	0	0	0	8
Total	250	160	1	23	5	439

### Fixation of femoral component

In total, there were 303 revision procedures where only the femoral component was revised. Again, a change towards cemented fixation was noted. Of 303 femoral revisions, 260 stems (86%) had been uncemented in primary THA (registered as uncemented and reversed hybrid), compared to 34 cemented (11%) stems (registered as cemented and hybrid). After revision surgery, 190 revised stems (62%) were uncemented, and 110 stems (36%) were fixated using bone cement (Table 6). In cases with a femoral revision procedure, the proportion of cemented fixation between primary and revision THA increased statistically significantly by 25% (CI 19–31; *p* < 0.001) (Figure 4).

**Table 6.** Method of fixation in primary and revision THA for stem revisions (*n* = 303).

Primary	Revision					
	Cemented	Uncemented	Hybrid	Reversed hybrid	Missing	Total
Cemented	18	9	0	0	0	27
Uncemented	64	160	11	0	2	327
Hybrid	2	5	0	0	0	7
Reversed hybrid	11	10	0	1	1	23
Missing	3	5	1	0	0	9
Total	98	189	12	1	3	303

### Survival of revision THA

**Figure 5.**
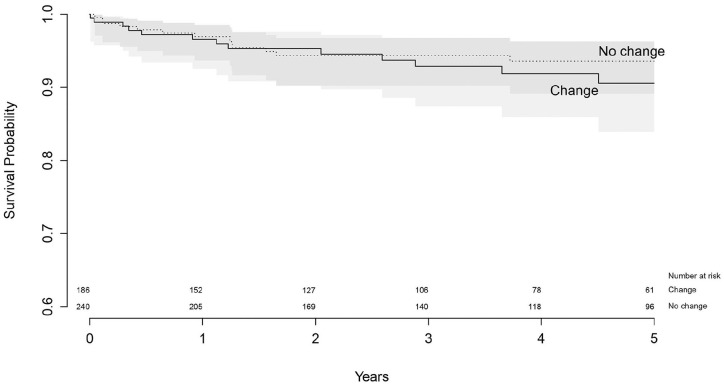
Survival of revised acetabular components with endpoint re-revision of the acetabular component.

Using Kaplan-Meier, the survival of both revised acetabular and femoral components was determined, with endpoint re-revision of the component, stratified for change in fixation method. For revised acetabular components that changed the fixation method, survival at 5 years follow-up was 90.6% (95% CI, 83.9–94.6), where survival of components that did not change the fixation method was 93.6% (89.1–96.3), which was not significantly different (*p* = 0.30) (Table 7) (Figure 5).

**Table 7.** Survival of revised acetabular- and femoral components by change in method of fixation.

	Method of fixation	2-year	5-year
Revised acetabular components	Change	95.3 (90.8–97.6)	90.6 (83.9–94.6)
	No change	94.4 (90.3–96.8)	93.6 (89.1–96.3)
Revised femoral components	Change	98.9 (92.3–99.8)	98.9 (92.3–99.8)
	No change	97.2 (93.5–98.8)	96.1 (91.3–98.3)

**Figure 6.**
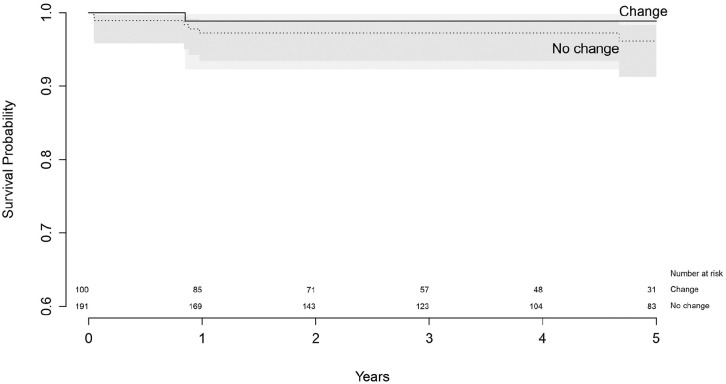
Survival of revised femoral components with endpoint re-revision of the femoral component.

For revised femoral components that changed the fixation method, survival at 5 years follow-up was 98.9% (95% CI, 92.3–99.8), where survival of components that did not change was 96.1% (95% CI, 91.3–98.3), which was not significantly different (*p* = 0.22) (Table 7) (Figure 6).

## Discussion

The aim of this paper was to assess differences in the fixation method used between primary and revision hip arthroplasty in young patients using data from the LROI. We found a remarkable increase in the use of cemented fixation in revision hip arthroplasty, when compared to primary THA, especially for the acetabular component.

Data on the long-term outcome of revisions in young patients are lacking, as only 2 reports are available. 1 report based on uncemented revision procedures in young patients <55 years showed disappointing results,^8^ whereas another study reporting on cemented revisions in young patients with bone impaction grafting showed more promising results.^9^ However, information based on large cohorts of young patients in which the type of fixation in revision surgery has been studied is lacking in literature.

As in most countries, in patients <55 years, most primary THA in the Netherlands were placed as full uncemented fixation (80%), followed by full cemented fixation (9%), reversed hybrid (8%) and hybrid fixation (2%). We found a remarkable increase in the use of cemented fixation in revision hip arthroplasty, when compared to primary THA, especially for the acetabular component. In acetabular only revisions, the use of cemented fixation increased statistically significantly by 39% (CI 34–45) from 23% in primary THA to 62% in revisions. The same pattern was seen at the femoral side, however, this increase was less pronounced. Cemented fixation in cases where the femoral component was exchanged increased significantly with 25% (CI 19–31), from 11% in primary THA, to 36% in revision THA.

As this study is an observational study using registry data, it is somewhat difficult to find explanations for this finding. The first explanation may be that cemented fixation in primary THA, especially in patients >70 years, is still regularly performed in the Netherlands. According to the LROI, 23% percent of primary THA are still cemented.^10^ Therefore, many Dutch orthopaedic surgeons still have the skills to perform a well-cemented hip implant. We tried to ascertain if the change in fixation could be explained by referrals of these younger patients to other centres for their revision procedure, as contrasting fixation policies in different hospitals could explain the observed trend towards cemented fixation. However, from all procedures with only an acetabular revision (*n* = 439), only 88 procedures (20%) were conducted in a different hospital compared to the primary procedure. From those 88 cases, 57 cases changed the fixation method. However, in cases where there was no change in hospital between primary and revision THA, still 1 out of 3 cases changed the fixation method (129 out of 338). From all procedures where only the femoral component was revised (*n* = 303), 41 cases were revised in a different hospital. From those 41 cases, 14 cases had a change in fixation method between the primary and revision THA. In the cases that did not change hospital, a similar number had a change in the fixation method (88 out of 262). Therefore, considering the large number of cases that changed the fixation method in the same hospital, we do not think this explains the change in the fixation method between primary and revision THA.

Secondly, the increase in the use of cemented fixation in revision hip arthroplasty might be explained by an increase in the use of bone grafts in revision procedures, as acetabular bone loss is a major concern for surgeons during revision hip arthroplasty in younger patients.^13,14^ Traditionally, bone impaction grafting on both the acetabular and femoral side has been popular in the Netherlands.^15,16^ However, registration of bone grafts was not incorporated within the LROI before 2014. Therefore, the effect of the use of bone grafts could not be explored for all years, as these data were not complete for all years of inclusion.

Thirdly, it might be expected that surgeons are more willing to change the fixation method when revising an early failure. Therefore, we analysed time to revision in procedures that changed or did not change the fixation method. For acetabular revisions, time to revision for cases that changed fixation was 3.6 years (SD 3.0), and 2.8 years (SD 2.5) for cases that did not change. For femoral revisions, time to revision in cases that changed was 2.5 years (SD 2.5), where time to revision in cases that did not change was 2.1 years (SD 2.4). For both acetabular and femoral revisions, mean time to revision for cases that changed the method of fixation was higher. Therefore, this does not explain the change in method of fixation between primary and revision THA.

For both the revised acetabular and femoral component, we found no statistically significant difference in the 5-year survival between revision procedures that changed or did not change the fixation method. Only a few cohort studies report on the effect of fixation type in revision procedures. For the acetabular side, Lie et al.^17^ found a statistically significant reduced risk of failure for uncemented revisions, where a study from the Swedish Hip Arthroplasty Register found no differences in survival between cemented and uncemented revised acetabular components.^18^ At the femoral side, uncemented fixation was associated with an increased risk of failure of revision procedures,^19^ where others found no effect of method of fixation on survival of revision procedures.^20,21^ However, some of these studies were single-centre studies with relatively low patient inclusion.^19,21^ None of these studies focused on young patients. Additionally, a change in method of fixation between primary and revision hip arthroplasty was not described. Although we found no difference in survival of revised components, more long-term data is needed to evaluate the effect of different methods of fixation on the survival of revision procedures.

There are a few limitations of this study that have to be considered, Firstly, the completeness of revision hip arthroplasty in the LROI is lower compared to the completeness of primary THA, especially in the first years of the registry, where there was no complete coverage of all Dutch hospitals.^11^ Therefore, we might have missed revision procedures in our analyses, which may have influenced our findings. Secondly, as already mentioned, the effect of the use of bone grafts could not be explored for all years, as registration of bone grafts within in LROI started in 2014. Lastly, as already described, there is no information within the LROI regarding the presence or magnitude of possible bone defects. Additionally, we did not analyse the use of any cup-cage constructions or triflange acetabular components in this study. Furthermore, the use of extended trochanteric osteotomies in revision hip arthroplasty is not registered within the LROI. All these factors might steer the surgeon in the decision to use cemented or uncemented fixation.

## Conclusion

We found an unexpected and significant change in fixation method between primary and revision hip arthroplasty in young patients in the Netherlands using a large dataset from the Dutch Arthroplasty Registry (LROI). There was a large increase in the use of cemented fixation during revision procedures. In cases where only the acetabular component was revised, cemented fixation even became the dominant method of fixation. No significant difference in short-term survival was found between revision procedures that changed or did not change the fixation method. Unfortunately, the possible clinical effect of fixation method on the outcome of revision hip arthroplasty is not clear at the moment, as the clinical follow-up is relatively short. Therefore, further studies are needed to evaluate this significant change in fixation method in young patients on the outcome of revision procedures.
